# Chronic Ulcers Healing Prediction through Machine Learning Approaches: Preliminary Results on Diabetic Foot Ulcers Case Study

**DOI:** 10.3390/jcm14092943

**Published:** 2025-04-24

**Authors:** Elisabetta Spinazzola, Guillaume Picaud, Sara Becchi, Monica Pittarello, Elia Ricci, Marc Chaumont, Gérard Subsol, Fabio Pareschi, Luc Teot, Jacopo Secco

**Affiliations:** 1 Department of Electronics and Telecommunications, Politecnico di Torino, 10123 Turin, Italy; elisabetta.spinazzola@polito.it (E.S.); sara.becchi@polito.it (S.B.); fabio.pareschi@polito.it (F.P.); 2 LIRMM, ICAR Team, University Montpellier, CNRS, 34000 Montpellier, France; guillaume.picaud@lirmm.fr (G.P.); marc.chaumont@lirmm.fr (M.C.); gerard.subsol@lirmm.fr (G.S.); 3 Associazione Italiana Ulcere Cutanee (A.I.U.C.), 10123 Torino, Italy; monicapittarello@hotmail.it (M.P.); eliaricci@tin.it (E.R.); 4 Site des Carmes, Univeristy of Nîmes Place Gabriel Péri, 30021 Nîmes, France; 5 Société Française et Francophone des Plaies et Cicatrisations, 91370 Verrieres Le Buisson, France; l.teot@cicat-occitanie.org

**Keywords:** diabetic foot ulcers, chronic wounds, machine learning, deep neural networks, predictive medicine, healing, multiclass segmentation

## Abstract

**Background:** Chronic diabetic foot ulcers are a global health challenge, affecting approximately 18.6 million individuals each year. The timely and accurate prediction of wound healing paths is crucial for improving treatment outcomes and reducing complications. **Methods:** In this study, we apply predictive modeling to the case study of diabetic foot ulcers, analyzing and comparing multiple models based on Deep Neural Networks (DNNs) and Machine Learning (ML) algorithms to enhance wound prognosis and clinical decision making. Our approach leverages a dataset of 1766 diabetic foot wounds, each monitored for at least three visits, incorporating key clinical wound features such as WBP scores, wound area, depth, and tissue status. **Results:** Among the 12 models evaluated, the highest accuracy (80%) was achieved using a three-layer LSTM recurrent DNN trained on wound instances with four visits. The model performance was assessed through AUC (0.85), recall (0.80), precision (0.79), and F1-score (0.80). Our findings indicate that the wound depth and area at the first visit followed by the wound area and granulated tissue percentage at the second visit are the most influential factors in predicting the wound status. **Conclusions:** As future developments, we started building a weakly supervised semantic segmentation model that classifies wound tissues into necrosis, slough, and granulation, using tissue color proportions to further improve model performance. This research underscores the potential of predictive modeling in chronic wound management, specifically in the case of diabetic foot ulcers, offering a tool that can be seamlessly integrated into routine clinical practice.

## 1. Introduction

Chronic wounds represent a significant and growing healthcare challenge worldwide. They are a syndrome that affects around 4% of the world’s population due to several pathologies [1,2]. In particular, wounds affect an estimated 8.2 million Americans per year, becoming more prevalent in the United States for multiple reasons, such as increasing percentages of obesity and diabetes [3]. One of the most common complications of diabetes is diabetic foot ulcers. They affect approximately 18.6 million people each year [4] and can lead to severe health consequences, including infections, amputations, and a substantial decline in quality of life. The wounds are difficult to heal and typically require prolonged treatment, placing a substantial burden on both patients and healthcare systems. According to the World Health Organization (WHO), diabetic foot ulcers are one of the leading causes of hospitalization in diabetic patients, emphasizing the need for better strategies to predict and manage healing.

**Figure 1:**
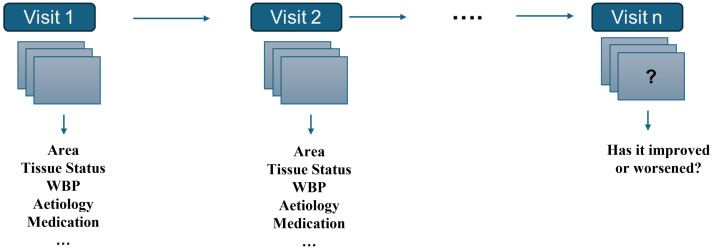
Healing prediction concept. Diagram of the process for monitoring a lesion across multiple visits (Visit 1, 2, …, n), in which parameters such as area, tissue status, WBP, and etiology are recorded. The data collected at each visit are compared to determine whether the patient’s condition is improving or worsening, thereby supporting prediction and treatment planning.

Chronic wounds do not have an ‘‘ordinary’’ healing time, defined as 4 to 12 weeks, and for this reason, it is difficult to assess proper treatments. Moreover, without proper or timely treatment, patients may face dire outcomes, including the loss of legs. A non-healing diabetic foot ulcer (DFU) results in an amputation every 30 s worldwide, with a 40–70% five-year mortality rate following amputation [5]. Consequently, medical costs are estimated to be up to 96.8 billion USD in 2014, and the annual wound care product market is estimated to reach 18.7 billion USD by 2027 [6]. In recent years, the need for predictive healing models in chronic wound care has gained increasing attention [7]. Healing prediction refers to the process of forecasting or estimating the progress and eventual outcome of a lesion healing, as well as in the context of wounds or general medical conditions. In particular, healing prediction involves the assessment of how well or how quickly a wound, injury, or condition will recover based on various factors, as described in Figure 1.

The ability to predict the healing path of diabetic foot ulcers could significantly improve patient recovery, reduce the risk of complications, and optimize the use of medical resources, preventing more severe consequences such as amputations.

Current state-of-the-art methods for predicting medicine and healing of chronic wounds focus primarily on clinical features, such as wound size, depth, medication, and the presence of infection. Berezo et al. developed gradient-boosted decision tree Machine Learning (ML) model using electronic health record (EHR) data to predict patients at risk of having wounds not heal within 4, 8, and 12 weeks from the start of treatment, achieving an AUC of 0.854 [3]. Horn et al. provide information regarding the creation of a risk-stratification system to predict the likelihood of the healing of body and heel pressure ulcers [8,9]. Other approaches concerns more specific Deep Neural Networks (DNNs) on different predictive medicine applications [10,11,12,13,14].

However, these methods lack applicability in highly specialized cases, such as diabetic foot ulcers, and do not to include complete data processing pipelines from wound identification and segmentation to healing prediction. The features extraction achieved from segmentation [15,16] contributes to wound healing prediction, underlying patient conditions, environmental factors, and treatment responses. Recent advancements in ML and computer vision have shown promising sophisticated models for wound healing prediction, yet there remains a gap in creating reliable systems for healing prediction in diabetic foot ulcers.

Instead, our proposal aims to fill this gap by providing a new predictive medicine model to create a robust framework for wound healing prediction. By combining the power of predictive analytics with clinical wound features, we aim to develop a system that not only forecasts healing outcomes but also provides advanced wound’s features. This approach focuses on a clinical case study tested on diabetic foot ulcers that shows promising and reliable results.

Moreover, in future perspective, we provide an ongoing work of building a weakly supervised semantic segmentation model. The model aims to classify wound tissues into necrosis, slough, and granulation by tissues color proportions. By incorporating new key features from the advanced segmentation, the final goal is to enhance the model’s performance.

The proposed system could potentially be integrated into the common good clinical practice and management of chronic wounds by providing tools that could help physicians to make informed, timely decisions, ultimately improving patient care and reducing the burden of chronic wound complications.

The remainder of the paper organized as follows. Section 2 starts with a brief overview of the device used in this project, the collected dataset, and the proposed methods. In Section 3, the paper provides a case study of the proposed methods on diabetic foot ulcers. Together, a future developments discussion on advanced segmentation technique is provided. In the end, the conclusion is drawn.

## 2. Materials and Methods

### 2.1. Wound Viewer

Some of the data used in this study were collected using a clinically validated wound imaging medical device designed to monitor the healing progress of chronic wounds. The Wound Viewer (WV) device, through a high-resolution digital camera, captures wounds during routine clinical visits. It provides precise clinical features of the wounds such as area, depth, etiology, tissue status, and more by employing customized Artificial Intelligence neuromorphic algorithms. The WV device has been developed due to the need to acquire wound images and classify them in an automated and precise way. The reliability of the device has been demonstrated through the results of the clinical trial with the protocol number OC 15194, identified by ethics committee approval of the Ethical Committee of the Azienda Ospedaliero-Universitaria San Luigi Gonzaga (Orbassano, Italy) [17,18,19].

**Figure 2:**
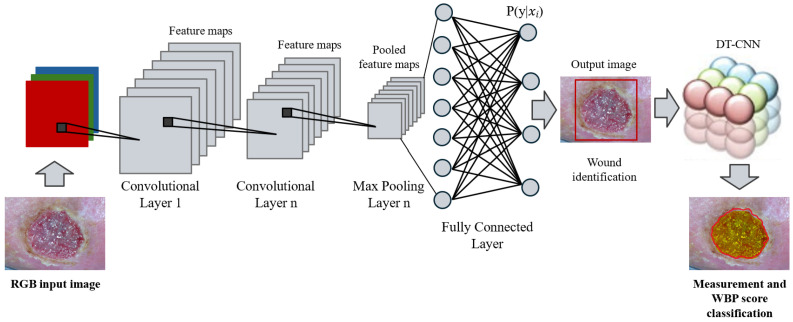
WV device algorithms and functionalities.The system detects the wound area, measures its size, and classifies tissue types for accurate monitoring. The figure is adapted from [17].

The device functionalities, shown in Figure 2, follow two steps, which correspond to two sub-networks. The first is responsible for image feature extraction, automatic wound detection, and ROIs identification through a multilayered convolutional neural network (CNN). The second block is instead composed of a Discrete-Time Cellular Neural Network (DT-CNN) based on the memristive cells of the Cellular Automata and the Belief Propagation Inspired algorithm. This second part provides a simple segmentation and subsequent classification of the wound based on the Wound Bed Preparation score (WBP).

Details regarding the camera conditions, lighting environment, and how these factors are normalized in our analytical pipeline have been carefully reported in [17], where the device is described.

### 2.2. Dataset

This study has been conducted through a dataset which consists of 5126 wounds. The dataset is composed of wound images and features acquired through the WV device during the previously mentioned clinical trial (protocol number OC 15194) and held by Politecnico di Torino in an anonymized fashion such that no information—neither textual, numerical, nor visual (e.g., images)—permits the re-identification of individuals in any form. The image database has also been extended with open-source datasets [20]. Therefore, there has been no direct human involvement for this study. The dataset includes patients with the broadest possible range of skin tones and characteristics in order to eliminate possible biases.

More dataset information are provided in the referenced articles [17,19], where the complete dataset and clinical trial description are reported. In particular, efforts were made in recruiting patients with different skin tones to ensure diversity. This data are made up of all the metadata and features extracted by the WV for at least three visits. The following features have been included: etiology; wound area; visit area; depth; infection; visit date; tissue status; WBP; exudate; and percentages of black, red, yellow, and white colors. Data are representative of patients with chronic wounds of different etiology, including diabetic foot ulcers.

### 2.3. Predictive Healing

Both ML algorithms and DNN models have been trained and tested for the mentioned scope. Before moving on to the characterization of the problem under examination, a brief general description of the tested models is provided. Different techniques have been explored to assess previously untested possibilities for the proposed task and to compare state-of-the-art approaches. For the ML techniques, we examined the following:

1. KNN (K-Nearest Neighbors): A simple, non-parametric classification algorithm. It works by assigning a data point to the most common class among its K-nearest neighbors. The distance between points is measured using Euclidean distance [21,22].
2. Random Forest: An ensemble learning method that builds a collection of decision trees during training and outputs the class that is the mode of the classes predicted by individual trees [23].
3. SVM (Support Vector Machine): A supervised algorithm used for classification and regression tasks. It works by finding the hyperplane that best separates data points from different classes. It maximizes the margin between the classes to achieve the optimal decision boundary [24].
4. Naive/Gaussian Bayes: A family of probabilistic algorithms based on Bayes’ Theorem, which assumes that features are conditionally independent given the class label. In the Gaussian Naive Bayes algorithms, the continuous features are assumed to follow a Gaussian (normal) distribution [25].
5. AdaBoost (Adaptive Boosting): An ensemble learning technique that combines multiple weak classifiers to create a strong classifier. It works by sequentially applying weak models to weighted versions of the training data, with the aim of correcting the errors made by previous models. AdaBoost adjusts the weights of misclassified data points so that subsequent classifiers focus more on these hard-to-classify points [26].
6. GradientBoost: An ensemble technique that builds a model in a stage-wise fashion by combining weak learners (typically decision trees) to form a strong predictive model. It works by fitting each new model to the residual errors made by the ensemble of previous models, thus “boosting” the performance iteratively [27].
7. XGBoost (Extreme Gradient Boosting) and LightGBM (Light Gradient Boosting Machine): Optimized implementations of gradient boosting, designed to improve both computational efficiency and model performance. XGBoost uses advanced regularization techniques and efficient handling of sparse data, while LightGBM focuses on speed and memory efficiency, particularly with large datasets [28,29].

Instead, among the DNN techniques, the following models were tested:

1. FCNN (Fully Connected Neural Network): Also known as Multilayer Perceptron (MLP), where each neuron in one layer is connected to every neuron in the next layer. It consists of an input layer, one or more hidden layers, and an output layer. FCNNs process input data through these layers by applying weighted sums, bias terms, and activation functions. They are commonly used for tasks like classification and regression. The network learns by adjusting its weights by backpropagation and the Learning Rate (LR) to minimize the error in its predictions [30].
2. LSTM (Long Short-Term Memory): A type of recurrent neural network (RNN) designed to model sequential time series of data. LSTMs can capture long-term dependencies by using memory cells that store information over extended periods of time. This capability is particularly useful for tasks that involve time-series data, where the model needs to remember previous inputs for accurate predictions [31].

#### Data Processing and Problem Design

Retrospective data from the WV dataset were used to train, validate, and test the models. The original dataset starts from 5126 wounds with at least 3 visits each, with more than 10 in some wound instances. In the context of predictive wound healing, given *N* visits, the primary goal is to predict whether the wound will improve or worsen by the subsequent visit (N+1). The lesion is monitored during the visits with different parameters, which are called features. In this case, the dataset provides a total of 13 features, of which are mentioned in Section 2.2.

For the data processing, the dataset has been rearranged in time series of wound instances, as will be shown in the case study in Section 3. Each time series is represented as a matrix which has a number of rows equal to the *N* visits and a number of columns equal to 13, which is the number of features. To assess the wound status at the N+1 visit, the chosen ML or DNN approaches use a supervised learning, which means that all the wound instances have a label associated to them as prediction output. The neural network learns how to associate the given output to each wound instance.

To build the labels, three data drivers, representing the most relevant clinical features, were used separately for each model: the WBP, the tissue status, and the exudate.

The label is determined by observing the evolution of the chosen data driver during the visits, from the first to the last, which we want to predict the status. The evolution is considered as the trend that indicates the improving or the worsening of the wound healing status. To assess the trend of each data driver, we evaluated all the possible data driver conditions. The WBP is a clinical assessment score used to evaluate the readiness of a wound bed for effective healing. The values are A, B, C, and D, which correspond to a progressively improving with granulation tissue (A) to worsening with necrotic tissue (D).

For the exudate, four values are possible: 0, 1, 2, and 3. These correspond to an inexistent exudate (0) to a huge amount of exudate (3).

In the end, the tissue status can be assessed by a good to bad healing with the following categories: Intact, Dry, Erithema, Hyperkeratotic, Macerated, or Cellulitis.

The assessed label of the wound instance can have only two possible outcome. The prediction of the healing status at the subsequent visit is a value that could be 1 if the wound is worsening or 0 if it is improving. These cases are defined as two possible classes of a binary classification problem.

The labels come from an algorithm, which evaluates the evolution of each data driver. Let us consider the labels extraction from the exudate. Define four visits values as $V_{1}$, $V_{2}$, $V_{3}$, and $V_{4}$; then, the following combinations have been treated as follows:

1. If $V_{4}$ > $V_{1}$ and $V_{4}$ > $V_{2}$ and $V_{4}$ > $V_{3}$ -> the label is 1, and the wound is worsening;
2. If $V_{4}$ < $V_{1}$ and $V_{4}$ < $V_{2}$ and $V_{4}$ < $V_{3}$ -> the label is 0, and the wound is healing;
3. If $V_{4}$ < $V_{1}$ and $V_{4}$ > $V_{2}$ and $V_{4}$ > $V_{3}$ -> the label is 1, and the wound is worsening;
4. If $V_{4}$ < $V_{1}$ and $V_{4}$ > $V_{2}$ and $V_{4}$ < $V_{3}$ -> the label is 0, and the wound is healing;
5. If $V_{4}$ < $V_{1}$ and $V_{4}$ < $V_{2}$ and $V_{4}$ > $V_{3}$ -> the label is 1, and the wound is worsening;
6. If $V_{4}$ > $V_{1}$ and $V_{4}$ < $V_{2}$ and $V_{4}$ > $V_{3}$ -> the label is 1, and the wound is worsening;
7. If $V_{4}$ > $V_{1}$ and $V_{4}$ > $V_{2}$ and $V_{4}$ < $V_{3}$ -> the label is 0, and the wound is healing;
8. If $V_{4}$ > $V_{1}$ and $V_{4}$ < $V_{2}$ and $V_{4}$ < $V_{3}$ -> the label is 0, and the wound is healing;
9. If a visit is equal to the previous or the next visit, both visits are considered ones and follow the same logic.

None of the database wound instances had visits with the four same values. The algorithm follows the main guidelines provided from experts, who also validated the output labels with the remaining features. In this way, the labels correspond to the clinical ground truth to train the models.

For the training of the models, the data are composed by the *N*-only visits series, omitting the features of the N+1 visit, to evaluate a prediction exclusively on patient’s clinical history.

From the data pre-processing point of view, we first performed a label encoding procedure of the features expressed by strings to standardize the dataset into numeric data.

Moreover, after a previous analysis, the amount of missing data was evaluated. When encountered, missing values were addressed by consulting domain experts to ensure accuracy and consistency. As for irregularly timed visits, these instances were excluded from the analysis. Prior to model development, the remaining data were chronologically ordered to preserve temporal consistency and reduce potential bias. This approach was adopted to enhance the reliability and validity of the model outcomes. For binary classification, it was necessary to balance the dataset, ensuring that the two classes were equally represented.

This procedure also ensures avoiding overfitting mitigation or potential sampling biases. In fact, especially in ML methods, imbalanced datasets can lead to biased models that perform poorly on the underrepresented classes, causing overfitting. A balanced dataset means that both classes (e.g., Class 0 and Class 1) have approximately the same number of samples. To implement this, data augmentation has been performed. The technique consists of measuring the overlapping between time series. It refers to creating new sequences by sampling parts of the data that share some of the same values. We used an overlapping window of 1 visit. Moreover, the features contain the visit date, which has been used to order the time series temporally. With the mentioned technique, the dataset achieved up to 5445 wound instances. Before training model, the dataset was randomly split into training (80%), validation (10%), and testing (10%) sets. In Table 1, all the performed experiments are shown.

A total of 12 ML models have been trained, tested, and validated. For each one, the architecture and the main parameters are provided. Each model has been trained and tested separately for each chosen data driver due to different label assessment. For each model with a specific data driver, the prediction has been performed on batch of time series of 3, 4, and 6 visits length. In this perspective, as mentioned previously, the dataset has been rearranged in time series of the following:

1. A total of 2 visits to predict the 3rd;
2. A total of 3 visits to predict the 4th;
3. A total of 5 visits to predict the 6th.

For each model with the corresponding data driver and time series batch, the performance outcomes were reported as the AUC obtained for the test set. AUC refers to the Area under the Receiver Operating Characteristic (ROC) curve.

This metric was the same used to train the model to obtain convergence.

**Figure 3:**
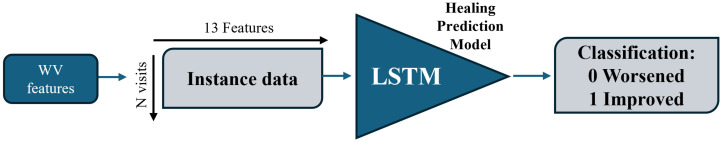
Proposed pipeline for feature extraction and final classification to predict lesion improvement or worsening.

**Figure 4:**
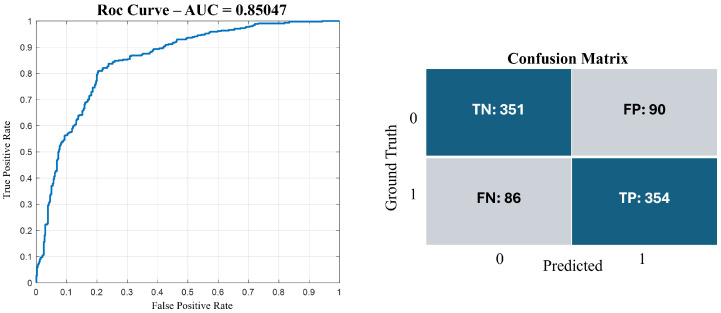
Performance evaluation of the proposed LSTM model using the ROC curve (AUC = 0.85047) and the confusion matrix. The results illustrate the model’s ability to discriminate between lesion improvement (0) and worsening (1).

As highlighted in Table 1, the best results were obtained with the exudate data driver in the batch of 4 visits. Among the tested models, the LSTM DNN achieved an 85% AUC as testing metric, which is a comparable result with similar state-of-the-art methods [3]. The predictive model framework for chronic wound healing is shown in Figure 3. The model performance was also assessed evaluating the precision, recall, accuracy, and F1-score metrics as follows:

(1) $$\mathrm{Precision}=\frac{\mathrm{TP}}{\mathrm{TP}+\mathrm{FP}};$$

(2) $$\mathrm{Recall}=\frac{\mathrm{TP}}{\mathrm{TP}+\mathrm{FN}};$$

(3) $$F1\;\mathrm{Score}=\frac{2*\mathrm{Precision}*\mathrm{Recall}}{\mathrm{Precision}+\mathrm{Recall}};$$

(4) $$\mathrm{Accuracy}=\frac{\mathrm{TP}+\mathrm{TN}}{\mathrm{TP}+\mathrm{TN}+\mathrm{FN}+\mathrm{FP}};$$

where TP, TN, FN, and FP are true positive, true negative, false negative and false positive, respectively. Accuracy, precision, recall, and F1-score are crucial metrics for evaluating the performance of classification models. In Figure 4, the confusion matrix and the ROC curve are shown, demonstrating accuracy of 80%, precision of 79%, recall of 80%, and F1-score of 80%.

For a medical point of view, these metrics help in understanding how well the model classifies the TP and TN. Overall, the model demonstrated balanced sensitivity and specificity (80% and 79%, respectively).

Moreover, the features importance has been evaluated. Feature importance evaluation is the process of assessing which features have the most influence on the predictive performance of an ML and DNN model. Understanding feature importance can help in model interpretation, feature selection, and overall optimization of ML systems. Our findings indicate that wound depth and wound area at the first visit are the two most influential factors, followed by wound area and red color percentage (granulated tissue) at the second visit.

**Figure 5:**
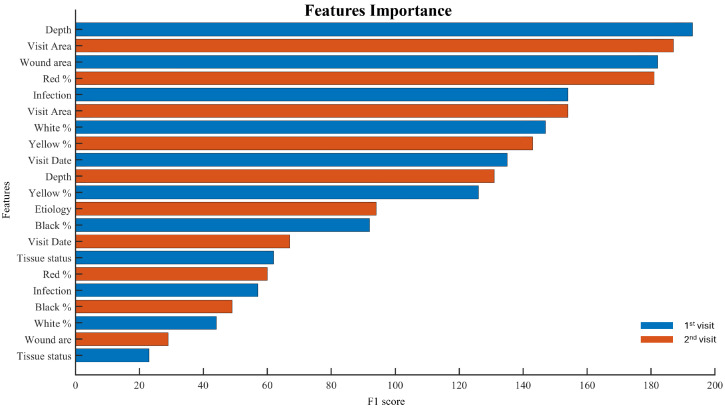
Feature importance analysis for the features measured during the first (blue bars) and second visit (orange bars). The horizontal axis shows the F1-score, indicating each feature’s contribution to the model, while the vertical axis lists only the features that proved to be relevant.

These results confirm the impact of the features extracted by the enhanced segmentation. All the feature importance evaluation is summarized in Figure 5.

## 3. Case Study: Diabetic Foot Ulcers

Diabetic foot ulcers are one of the represented etiologies in the dataset used. Given its impact and importance, we explored the model performance on this specific category of wounds. Healing prediction has been applied on 1.766 diabetic foot ulcers data through the proposed analysis. In this case study section, we aim to provide valuable insights, showing the visual and numerical results of the presented models’ performance.

### 3.1. Prediction Healing

For the specific case of diabetic foot ulcers, three types of wounds were included for this case study analysis: ’Chronic—Diabetic foot—Mixture’, ’Chronic—Diabetic foot—Neurological’, and ’Chronic—Diabetic foot—Vascular’. Here, two model outcomes are reported as examples of the healing predictions and the features which characterize the wound instances.

**Figure 6:**
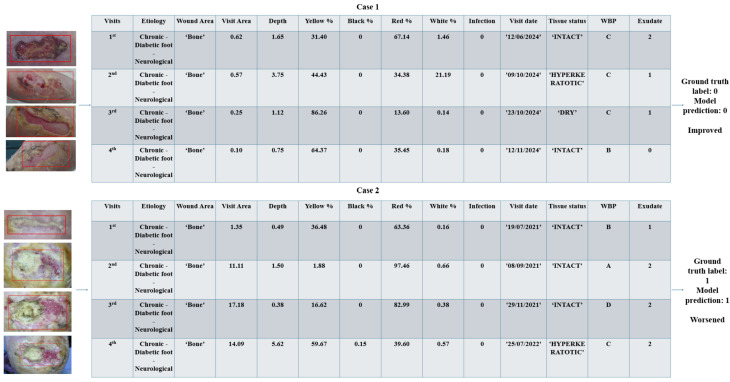
Predictive healing case study: two specific cases are presented—one where the model predicted improvement (**top**) and one where it predicted worsening (**bottom**). The tables show the features provided as input to the model, along with the corresponding ground truth labels and the model’s predictions.

In Figure 6, Case 1 involves the output prediction performed on an improved wound, while the Case 2 represents a sample of a worsened wound. The instances are represented with the time series of features and images for a batch of four visits. The first case involves a diabetic foot ulcer that initially had a larger area of 0.62 cm^2^ but showed promising signs of healing over time.

**Figure 7:**
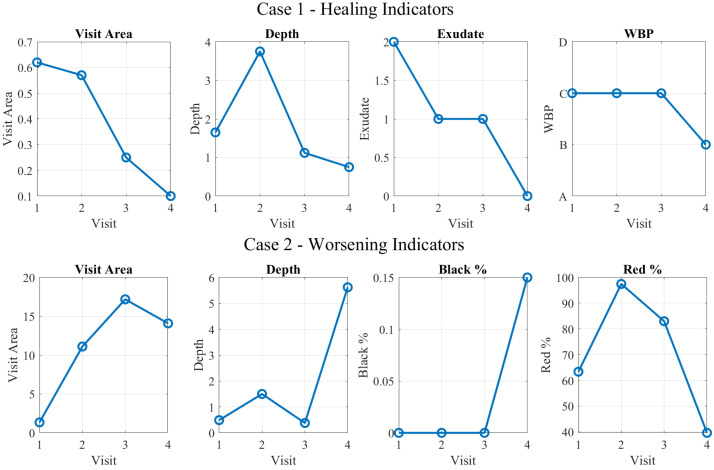
Predictive healing case study. Case 1: Trend of parameters contributing to healing (**top**). Case 2: Trend of parameters contributing to worsening (**bottom**).

Observing the features changing over visits in Figure 7, Case 1, where the wound area decreased, a sign of effective healing and tissue regeneration is shown by the exudate decreasing and WBP improving, significantly indicating that the wound is healing, with reduced inflammation and improved circulation. These features are characteristic of a positive healing path, suggesting that continued conservative care and proper wound management, such as appropriate dressings and infection prevention, will likely lead to complete healing.

On the other hand, the second case presents a moderate-sized diabetic foot ulcer with an initial small wound area. Over time, as shown in Figure 7, Case 2, the wound area increased to 11.11 cm^2^, indicating worsening. In addition to the enlargement of the wound, the color percentage of the wound showed a significant changes: the percentage of black color increased, while the red decreased. The increases in the area, depth, exudate, and black percentage are key indicators of wound deterioration, requiring aggressive intervention, such as debridement and infection control, to prevent further deterioration. In the end, the model predictions match with the ground truth labels for both cases.

From an overall perspective, the ground truth labels on diabetic foot ulcers are balanced in percentage of both classes. The model output demonstrated significant reliability of prediction, showing 87.5% for its AUC. The performance reveals a tendency to predict more cases of worsening rather than improving, suggesting a precision of 100% for the improved cases and 80% for the worsened cases. The recall and the F1-score were 100.0% and 88.89% for the worsening class, while they were 75% and 85.71% for the improving class, respectively. With the reported results, we can assess that given that the model predicts more worsening cases than healing cases, it is likely to be more sensitive rather than specific.

Sensitivity refers to the recall and to the model’s ability to correctly identify positive cases (in this case, worsening ulcers). Specificity refers to the ability of the model to correctly identify negative cases (in this case, improved ulcers).

In summary, the model appears to be more sensitive, meaning it is good at identifying worsening ulcers, but it may sacrifice some specificity, predicting some improving ulcers as worsening ones. From a clinical point of view, a higher sensitivity is generally preferred, especially in the context of the treatment assessment of diabetic foot ulcers, due to the fact that the early detection of worsening conditions is fundamental for timely intervention.

This observation may stem from the nature of the data used for training, where cases of worsening or non-healing ulcers might be more prevalent or exhibit more distinct features compared to those that heal. However, this also highlights limits and the need for further refinement in the model, such as incorporating additional factors, to achieve a more balanced prediction. Ensuring that the model can accurately predict both healing and worsening cases with similar reliability will be crucial for optimizing its effectiveness in guiding clinical decision making.

### 3.2. Discussion

The prediction of wound healing time based on a maximum of four clinical visits represents a promising direction for future research. Observing the wound progression within this structured time frame allows for the early identification of healing trajectories, which is particularly valuable in clinical decision making. This approach emphasizes the temporal evolution of wound characteristics and can help differentiate between faster and slower healing patterns. By analyzing changes across multiple visits, models can capture the dynamic nature of wound healing, potentially leading to more accurate and personalized prognoses. Further studies focusing on longitudinal trends and incorporating temporal modeling are likely to yield insights and strengthen predictive performance. Several aspects have also emerged in this previous analysis. These are linked to both the limitations and future steps which need to be addressed in order to improve the whole framework.

From the features importance analysis, future approaches will focus on the use of only a few top-ranked features as model inputs. The state of the art suggests this type of approach in support vector classification (SVC) models, trained using the seven highest-ranked individual features, achieving an AUC of 0.87 [32]. Until now, we trained the models using smaller inputs, observing a decrease in performance. Based on our experience, the features importance effectively highlights the key features that contribute most to the classification task. However, despite their high individual relevance, the overall performance of the classification model tends to improve when a larger number of input features are included. This may be attributed to several factors. Additionally, in an attempt to reduce the input dimensionality, Principal Component Analysis (PCA) has been applied as a pre-processing step. However, this approach did not lead to any improvement in model performance.

Potential differences in models accuracy could also be discussed across ulcer types. As a preliminary evaluation, we compared the model performance in forecasting healing times for diabetic foot ulcers and pressure ulcers. The results suggest that the predictive accuracy may vary between the two (AUC = 87.5% and 85%, respectively), which is likely due to their distinct underlying pathophysiological mechanisms, progression rates, and clinical presentation. For instance, diabetic foot ulcers often exhibit more complex vascular and neuropathic components, which may introduce greater variability in healing patterns compared to pressure ulcers. These differences could influence the relevance and weight of predictive features across ulcer types, warranting further investigation and potentially ulcer-specific model optimization.

Regarding the role of unique data drivers to assess labels, these intermediates may not accurately reflect meaningful clinical endpoints. The use of a key feature to assess a label is a methodological need to create a classification machine, which in the end revealed a reliable classification that was also justified by the evolution of the input features. Therefore, elements like complete healing, infection, full epithelialization, hospitalizations, amputations, and so on were included in the remaining features, which were given as input of the classification machine. In the end, the proposed method is a proof of concept that demonstrates that the technique could produce a reliable classification, even if more steps and integrations are necessary to provide a complete clinical overview.

As the computational overhead concerns, particularly for the LSTM model, the computational costs are an important consideration for a future implementation. Currently, the system is still in its preliminary stages, and further investigations are necessary to fully assess its computational efficiency. Regarding the current system, the data elaboration was performed on a cloud server, where local computational overhead is not a significant constraint. We report that the additional LSTM model for healing prediction has a total number of parameters equal to 568,800, which is a small value compared to more complex DNNs. Regarding the inference time, the algorithm takes 50 ms to perform an inference on a local PC CPU. However, it is important to note that the data processing and model inference will be performed on the same cloud server, which should help mitigate local computational overhead. As development progresses, we plan to provide more detailed information on the computational requirements in future iterations.

Future external validation with new datasets will be also necessary to assess generalization. It is also important to note that the validation with different datasets is limited by their heterogeneity. Unfortunately, there is no standardization for wound dataset features, and this issue is further compounded by the restricted public accessibility of these datasets.

Translating the outcomes of this research into clinical workflows holds strong potential for supporting decision-making processes in wound management. By leveraging predictive insights from early clinical visits, clinicians could be equipped with timely and individualized assessments of healing trajectories, allowing for more informed treatment planning and resource allocation. While the current study primarily focuses on model development and validation, the integration of these predictive tools into real-world practice would benefit greatly from the development of user-friendly interfaces or dashboards. Such tools would enhance the interpretability and accessibility of model outputs for clinical staff, supporting adoption without requiring technical expertise. Although interface design is beyond the current scope, we acknowledge its importance and view it as a key direction for future work aimed at fostering clinical utility and promoting seamless integration into healthcare systems.

### 3.3. Future Directions: Enhanced Wound Segmentation

Future research could explore healing prediction for other types of chronic wounds represented in different databases. For instance, the CICAT-Occitanie database contains 130,000 photographic images of chronic wounds collected over more than 15 years by experts as part of the telemedicine project “Domoplaies” [33,34]. This database is distinguished by its diversity in acquisition devices (smartphones operated by different nurses), by variations in scenes and conditions (shooting angles, distances between the smartphone and the wound, lighting conditions, and backgrounds), as well by a wide range of wound types and anatomical locations. These images are associated with proportions for three types of wound tissues: necrotic tissue, slough tissue, and granulation tissue. Those proportions were visually estimated by an expert, without any measurement tools, and were quantified with a precision of 10%. A multiclass semantic segmentation method capable of distinguishing between necrosis, slough, and granulations tissues is currently under study. This model is being trained in a weakly supervised manner, learning from the available proportion annotations to precisely differentiate tissue types. A potential direction could be to integrate this feature extractor into the methodology developed in this paper. As suggested by the results of the case study, the color percentages reveal relevant features in the prediction of healing assessment. These features are strongly linked to the advanced wound segmentation model, which could provide new elements to insert into the proposed prediction model.

**Figure 8:**
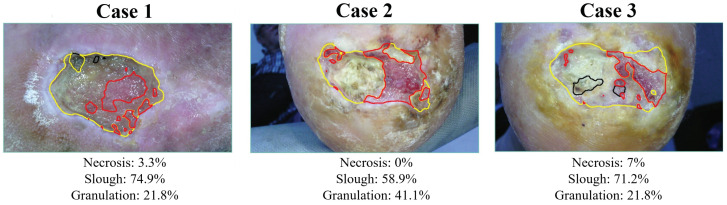
Multiclass classification for wound tissues composition with the relative segmentation masks and tissues proportions.

This connection is evident in Figure 8, where the first results obtained by the segmentation model are shown. In particular, it is clear how the tissue classification is partially determined by the wound color percentages. In this perspective, the wound tissue classification provided by the enhanced segmentation could improve the healing prediction accuracy with the introduction of advanced features.

## 4. Conclusions

This study demonstrates the significant potential of predictive modeling in the management of chronic diabetic foot ulcers, highlighting the ability of advanced ML and DNN approaches to enhance clinical decision making. By exploiting clinical features and advanced segmentation of wound tissues, we developed a robust model that accurately predicts wound healing outcomes, offering valuable insights for clinicians. The high performance of the three-layer LSTM recurrent DNN, achieving an accuracy of 85% and a favorable balance of AUC, precision, recall, and F1-score, emphasizes the relevance of wound depth and visit area at the first visit followed by the wound area and granulated tissue percentage at the second visit.

These findings represent a step forward in our understanding of wound prognosis and provide a foundation for integrating predictive analytics into routine clinical practice, with the ultimate aim of improving patient outcomes and reducing complications associated with diabetic foot ulcers.

Future research could focus on refining these models with larger, more diverse datasets and exploring their applicability across different patient populations.

## Figures and Tables

**Table 1:** Trained and tested models description and respective accuracy performance in AUC percentage.

		Models Accuracy								
		**N. Visits: 3**			**N. Visits: 4**			**N. Visits: 6**		
**Model**	**Architecture**	**WBP**	**Exudate**	**Tissue Status**	**WBP**	**Exudate**	**Tissue Status**	**WBP**	**Exudate**	**Tissue Status**
LSTM	^1^	55%	61%	49%	71%	80%	49%	61%	75%	50%
LSTM	^2^	66%	60%	50%	70%	85%	56%	65%	75%	51%
FCNN	^3^	42%	54%	61%	63%	62%	44%	59%	75%	52%
KNN	^4^	62%	48%	53%	73%	81%	55%	65%	70%	52%
Random Forest	^5^	60%	62%	54%	76%	71%	55%	71%	72%	55%
SVM	^6^	51%	58%	50%	78%	80%	55%	70%	70%	55%
Naive Bayes	^7^	39%	48%	56%	72%	81%	49%	70%	71%	50%
Gaussian Naive Bayes	^8^	61%	41%	49%	78%	82%	55%	71%	70%	51%
AdaBoost	^9^	55%	60%	56%	75%	82%	55%	72%	73%	52%
Gradient Boost [3]	^10^	59%	60%	55%	70%	79%	47%	71%	75%	53%
XGBoost	^11^	62%	58%	59%	77%	79%	55%	70%	75%	51%
LightGBM	^12^	61%	58%	56%	79%	80%	58%	71%	71%	53%

^1^ RNN with 1 layer of 200 hidden units; Adam optimization; 0.002 LR; AUC metrics. ^2^ RNN with 3 layers of 200 hidden units; Adam optimization; 0.002 Initial LR; AUC metrics. ^3^ Total of 4 fully connected layers and ReLU activation function; Adam optimization; 0.002 LR; AUC metrics. ^4^ Classification algorithm with 20 K-neighbors and Euclidean distance metric. ^5^ Ensemble learning with 300 trees. ^6^ Supervised algorithm with Radial Basis Kernel Function and 25 K-folds. ^7^ Probabilistic algorithm with kernel distribution. ^8^ Probabilistic algorithm with normal distribution. ^9^ Ensemble learning with AdaBoostM1 method. ^10^ Ensemble technique with LogitBoost method. ^11^ Optimized gradient boost with binary logistic objective. ^12^ Optimized gradient boost with binary objective.

## Data Availability

The dataset from Politecnico di Torino is available upon reasonable request (contact: elisabetta.spinazzola@polito.it). The data from CICAT-Occitanie database are available upon reasonable request (contact: guillaume.picaud@lirmm.fr).

## Abbreviations

The following abbreviations are used in this manuscript:

EHR	Electronic Health Record
ML	Machine Learning
DNN	Deep Neural Network
WV	Wound Viewer
LSTM	Long Short-Term Memory
RNN	Recurrent neural network
FCNN	Fully Connected Neural Network
KNN	K-Nearest Neighbors
SVM	Support Vector Machine
LR	Learning Rate
WBP	Wound Bed Preparation
ROC	Receiver Operating Characteristic
AUC	Area Under Curve
TP	True Positive
TN	True Negative
FN	False Negative
FP	False Positive
